# Development and Validation of the Interpreting Learning Engagement Scale (ILES)

**DOI:** 10.3390/bs15010016

**Published:** 2024-12-28

**Authors:** Wenting Yu, Chenggang Wu

**Affiliations:** 1 School of English Studies, Shanghai International Studies University, Shanghai 201620, China; wenting_yu@shisu.edu.cn; 2 Key Laboratory of Multilingual Education with AI, School of Education, Shanghai International Studies University, Room 1332, 1550 Wenxiang Road, Songjiang, Shanghai 201620, China; 3 Institute of Linguistics (IoL), Shanghai International Studies University, Shanghai 201620, China; 4 Shanghai Key Laboratory of Brain-Machine Intelligence for Information Behavior, Shanghai International Studies University, Shanghai 201620, China

**Keywords:** interpreting learning engagement, behavioral engagement, emotional engagement, cognitive engagement, agentic engagement

## Abstract

This study developed and validated the Interpreting Learning Engagement Scale (ILES), which was designed to measure the engagement of students in the interpreting learning context. Recognizing the crucial role of learning engagement in academic success and the acquisition of interpreting skills, which demands considerable cognitive effort and active involvement, this research addresses the gap in empirical studies on engagement within the field of interpreting. The ILES, comprising 18 items across four dimensions (behavioral, emotional, cognitive, and agentic engagement), was validated with data collected from a cohort of 306 students from five universities in China. The study employed exploratory and confirmatory factor analyses to establish the scale’s theoretical underpinnings and provided further reliability and validity evidence, demonstrating its adequate psychometric properties. Additionally, the scale’s scores showed a significant correlation with grit, securing the external validity of the ILES. This study not only contributes a validated instrument for assessing student engagement in interpreting learning but also provides implications for promoting engagement through potential interventions, with the ultimate aim of achieving high levels of interpreting competence.

## 1. Introduction

Amidst the growing acknowledgment of the pivotal role learning engagement plays in fostering academic success (Fredricks et al., 2004; Reschly & Christenson, 2022; Skinner et al., 2009), the educational research community has witnessed a surge in interest and scholarly endeavors surrounding this concept over the past two decades (Sinatra et al., 2015; Zhou et al., 2021). Learning engagement, delineating the degree of a student’s active involvement in a learning task and the purposefulness of their physical and mental exertion, has emerged as a focal point (Hiver et al., 2024). The attainment of high levels of learning engagement is associated with various favorable educational outcomes, including heightened academic persistence and enhanced effort, achievement, and aspirations, as well as improved mental well-being. Additionally, it correlates with reduced dropout rates and mitigates high-risk behaviors (Reschly & Christenson, 2022). Many stakeholders concur that engagement serves as a pivotal leading indicator of academic performance and eventual attainment (Mercer, 2019; Philp & Duchesne, 2016; Zhou et al., 2021).

Engagement is vital in various educational contexts, including interpreting training and learning. In the realm of interpreter training, the demanding nature of cross-linguistic tasks and the multifaceted skill set required for optimal performance necessitate substantial cognitive effort (Moser-Mercer, 2008). Indeed, interpreting learning requires learners not just going through the motions of practice but expending focused energy, attention, and active participation and being emotionally involved. Despite the pivotal role of engagement in enabling interpreters to effectively navigate complex interpreting scenarios in real-world settings (Moser-Mercer, 2008), learning engagement has received relatively scant attention in empirical studies compared to other personal factors pertinent to interpreting competence, such as language proficiency, working memory capacity, and executive control (Dong, 2018; Johnson, 2017). While prevailing psychological approaches to interpreting studies have primarily focused on three well-established factors—motivation, self-efficacy, and anxiety (Cai et al., 2023)—research on the psychological construct of engagement remains notably sparse. Therefore, it is both reasonable and pertinent for the interdisciplinary field of interpreting to leverage the extensive body of knowledge in educational psychology and explore engagement as a pivotal psychological factor in interpreter learning.

Specifically, given the significant role engagement seems to play in the learning process of students across various contexts and within numerous learning subdomains, there is an urgent need for reliable, valid, and domain-specific measures of student engagement (Zhou et al., 2021). Presently, the predominant method used to evaluate engagement is through self-report survey instruments (Zhou et al., 2021), yet research on interpreting learning engagement remains conspicuously absent. Against this backdrop, this study endeavors to address this gap by striving to adapt and validate an engagement scale tailored for interpreting learners, known as the ILES. This scale should function as a tool for evaluating not only the quantity but also the quality of interpreting learners’ active participation and engagement. By mirroring its application in students’ learning processes, it can serve as an indicator of their learning motivation and achievement. The ILES may also shed light on the promotion of interpreting learners’ engagement through intervention.

## 2. Literature Review

### 2.1. Learning Engagement and Language Learning

As the key to addressing problems of low achievement, high levels of student boredom, alienation, and high dropout rates, engagement has received growing recognition for its importance in contemporary education, leading to an exponential increase in popularity with researchers, educators, and policymakers (Fredricks et al., 2004; Sinatra et al., 2015). Engagement is an important educational outcome in its own right as a marker of students’ positive functioning, but it is further important because it predicts highly valued outcomes, such as a student’s academic progress and achievement (Ladd & Dinella, 2009; Skinner et al., 1998). Furthermore, engagement is considered malleable and responsive to intervention, which draws attention from all sides as evidence builds for promoting engagement across social and academic contexts (Appleton et al., 2008).

Whilst research on engagement has increasingly captivated the attention of educators and educational psychologists, it has become especially relevant for scholars and practitioners within the domain of language learning (Hiver et al., 2024; Oga-Baldwin, 2019). Second and foreign language (L2) learning in instructed settings requires deliberate and sustained involvement from learners, and engagement has become a popular framework for L2 researchers seeking to understand how learners proactively attend to their L2 learning, interact with peers and others while learning, and perform in diverse L2 classroom activities and settings over time (Zhou et al., 2021).

Despite the considerable variation in how the construct of engagement has been conceptualized over time (Appleton et al., 2008; Fredricks et al., 2004; Jimerson et al., 2003), a general consensus has emerged to characterize engagement in a language class as a dynamic, four-component construct, defined in the previous literature as behavioral, emotional, cognitive, and agentic engagement (Oga-Baldwin, 2019; Zhou et al., 2021). Behavioral engagement refers to the amount and quality of learners’ in-class participation (Reschly & Christenson, 2006). Examples of behavioral engagement in L2 learning include learners’ voluntary involvement in speaking, interactional initiative, time on task, the amount of semantic content produced while on task, and persistence on task without the need for support or direction (Philp & Duchesne, 2016). Emotional engagement in L2 instructional settings is often manifested in learners’ personal affective reactions as they participate in target language-related activities or tasks. Enthusiasm, interest, and enjoyment, which are typical markers of one’s affective involvement during class time, have been identified as critical indicators of emotional engagement in the classroom (Skinner et al., 2009). Cognitive engagement refers to mental processes such as the deliberate allocation and maintenance of attention and intellectual effort. In L2 classroom settings, research on cognitive engagement has focused primarily on verbal manifestations, including peer interactions, asking questions, volunteering answers, exchanging ideas, offering feedback, providing direction, informing, and explaining (Hiver et al., 2024). Agentic engagement corresponds with how learners contribute to the learning environment and the quality of instruction (Jang et al., 2016; Reeve & Tseng, 2011). Prominent both within and outside of language classrooms (Philp & Duchesne, 2016), this engagement dimension captures the process in which students intentionally and somewhat proactively try to personalize and otherwise enrich both what is to be learned and the conditions and circumstances under which it is to be learned (Reeve & Tseng, 2011).

The various facets of engagement are interconnected, with each one leading to changes in the others. On the other hand, however, engagement is not static or immutable. It is potentially malleable through contextual factors, such as teacher instructional styles and teacher and peer relationships, that can be targeted in interventions (Appleton et al., 2008; Fredricks et al., 2004). Hence, accurate and effective measurement of engagement can be used to guide intervention efforts aimed at improving student engagement. Among the various approaches to measuring engagement, self-report survey instruments have become the most frequently used tools (Oga-Baldwin, 2019; Sang & Hiver, 2021) due to their practical advantages, such as ease of administration, efficiency, and suitability for large classroom settings (Dörnyei & Taguchi, 2009). Self-reports are particularly useful for assessing dimensions like emotional and cognitive engagement, which are less observable through external behaviors (Appleton et al., 2006; Garcia & Pintrich, 1996). However, the limitations of self-report surveys must also be acknowledged. These include potential biases from participants, who may consciously or unconsciously provide responses that do not accurately reflect their actual engagement levels (Rosenman et al., 2011). Alternative methods, such as direct observation, expert ratings, interviews, and experience sampling, can offer more objective or real-time insights into student engagement. Nevertheless, logistical constraints make these methods challenging to implement with large-scale samples. For example, observational methods allow researchers to capture nuanced behaviors and interactions in the classroom but are time-consuming and require significant training for observers (Bakeman & Quera, 2011). This study employed self-report surveys for measuring engagement, primarily due to their efficiency and feasibility in the given research context. Nonetheless, the limitations of this approach are recognized, and future studies are encouraged to adopt a mixed-methods design that combines self-reports with observational or physiological measures (e.g., eye-tracking and biometric data). Such triangulation could provide a more comprehensive understanding of learning engagement, addressing the shortcomings of any single method. Some of the most prevalent self-report instruments have been pioneered, refined, and well-publicized (Jang et al., 2016; Reeve, 2013; Reeve & Tseng, 2011; Skinner et al., 2008; Skinner & Belmont, 1993). These survey instruments manifest some or all of the four sub-components of engagement in language learning listed above. The items pertaining to these sub-components and their psychometric functioning are well delineated in these studies, based on which Oga-Baldwin (2019) provided a summary of a collection of this line of research, and synthesized the commonly used items that measure behavioral, emotional, cognitive, and agentic engagement.. These items were applied to language learning (listening and speaking) by Oga-Baldwin and colleagues (Oga-Baldwin & Fryer, 2018; Oga-Baldwin & Nakata, 2017; Oga-Baldwin et al., 2017).

### 2.2. Learning Engagement and Its Measurement in Interpreting Learning

As a cross-linguistic activity that involves listening (to the source language) and speaking (in the target language), interpreting is cognitively more demanding than L2 oral tasks, which account for only part of the interpreter’s overall activity. Seeber (2011, 2015) pointed out that the inherent capacity limitations of the human cognitive system are continuously tested during interpreting tasks. What distinguishes interpreting from everyday spoken language activity is readily appreciated by basing the comparison of the two on models often used to analyze them: Levelt’s (1993) speech production model and Gile’s (2009) Effort Models of interpreting. The main difference between the two is that the speech production model has an initial conceptualization stage, whereas interpreting starts with perception and comprehension of the source language, with parallel storage, processing, and retrieval of information through note-taking, memory functions, and the coordination of all these efforts (Yu & Van Heuven, 2017). As a result, more attentional resources and cognitive loads (Seeber, 2011, 2015; Seeber & Kerzel, 2012) are almost certainly required in interpreting than in spontaneous speech production. Moser-Mercer (2008) suggests that achieving high levels of interpreting performance requires considerable effort. As an interpreting trainer and researcher, she admits that interpreting trainers often find themselves at a loss as to how to quantify the amount of effort involved in the acquisition of the skill of interpreting (Moser-Mercer, 2008). More recently, Dong (2018) explored interpreting training as a dynamic system and argued that developing interpreting competence involves great efforts in coping with limited working memory capacity and high-pressure performance environments. Here, a fundamental consideration with a view to measuring interpreting learners’ engagement with interpreting training that leads to their final accomplishment of this cross-linguistic skill is the importance of developing an engagement scale for interpreting learning. So far, no interpreting research has emulated studies of language learning in generating an effective engagement scale in the specific domain of interpreting learning. Such an attempt is potentially of considerable significance as a basis for guiding intervention methods to retain, boost, or regain students’ engagement in the process of interpreting learning. In this study, we attempted to adapt Oga-Baldwin’s (2019) Engagement Scale Items for Language Classrooms to the interpreting learning setting and validate such a scale.

Given the well-established link between grit and language learning (Wu et al., 2024), our study incorporated general grit as an external variable presumed to influence interpreting learning engagement. Grit, as conceptualized by Duckworth & Quinn (2009), embodies sustained effort and passion directed toward a specific goal. It encompasses two key components: perseverance of effort and consistency of interests. Interpreting is inherently cognitively demanding, requiring substantial skill refinement and persistent effort to master. Therefore, we hypothesized that grit would exert a significant influence on interpreting learning outcomes.

## 3. Methods

### 3.1. Participants

The study involved a cohort of 306 students enrolled in foreign language studies or translation and interpreting programs, consisting of 49 males and 257 females, with an average age of 20.79 years. These participants were selected from convenience samples recruited from five universities, which are located in cities across North, East, South, and Central China, including Beijing, Shanghai, Xiamen, and Jiaozuo. Recruitment took place from Consecutive Interpreting classes at each university, with participants invited to participate during scheduled class time with the approval of their course instructors. The sample comprised students across various academic levels. In the Chinese education system, freshmen, sophomores, juniors, and seniors correspond to first- to fourth-year undergraduate students. Graduate students are enrolled in master’s programs, with first-, second-, and third-year classifications indicating their respective years of study. The sample included freshmen (3.3%), sophomores (23.2%), juniors (13.4%), seniors (27.5%), first-year graduate students (18.6%), second-year graduate students (11.4%), and third-year graduate students (2.6%). Among the participants, the most prevalent sub-groups were undergraduate students majoring in foreign language studies (38.9%) and translation and interpreting (30.7%), alongside graduate students in non-academic track translation and interpreting programs (21.2%). This distribution was consistent across academic levels. The participants’ interpreting language pairs involved five combinations: Chinese–English (88.2%), Chinese–Japanese (7.8%), Chinese–Arabic (2.3%), Chinese–Russian (1.3%), and Chinese–Indonesian (0.3%). All participants were native Chinese speakers, with the other languages studied as foreign languages.

### 3.2. Measurement

In order to develop and validate the ILES (both internal consistency and external consistency with another psychological construct, i.e., grit), an online questionnaire was designed and structured into three sections. The first section covered the individual’s demographic information and interpreting learning experiences. The second section was the Interpreting Learning Engagement Scale (ILES), which consists of four dimensions with nineteen items in total: behavioral engagement (BE, five items), emotional engagement (EE, four items), cognitive engagement (CE, five items), and agentic engagement (AGE, five items). The original items were retrieved from one recent review on the engagement of language learning (Oga-Baldwin, 2019). The wording has been adapted to fit into interpreting learning classrooms (e.g., BE: “I participated in the activities of this semester’s interpreting classes.”; EE: “I felt good in this semester’s interpreting classes.”; CE: “I tried to fully comprehend the exact messages in the source speeches in this semester’s interpreting classes.”; and AGE: “I asked questions to help myself learn more knowledge and skills of interpreting in this semester’s interpreting classes.”). The construct was also affirmed by two experts on interpreting teaching. The ILES is a five-point Likert scale ranging from 1 (completely disagree) to 5 (completely agree). A higher score indicates higher engagement of interpreting learners. The third section was the Short-Grit Scale (Grit-S), which contains two dimensions with eight items: consistency of interest (CoI, four items) and persistence of effort (PoE, four items). Grit-S, which was used to investigate the external validity of the ILES, was translated from the original Short-Grit Scale developed and validated by Duckworth and Quinn (2009). Participants responded to eight items on a five-point Likert scale ranging from 1 (completely disagree) to 5 (completely agree). Items in PoE were regular-scored, while those in CoI were reversely coded. A higher score represents a higher level of grit.

All the measures in the second and third sections of the questionnaire were adapted and/or translated into Chinese following standard assessment translating procedures (Brislin, 1970; Hambleton & Lee, 2013). Initial translation of the ILES and Grit-S was performed by two functionally bilingual translators. Subsequently, an American graduate student, who is proficient in both Chinese and English, blindly translated the items back into English. The final translated versions were compared with the original versions of the ILES and Grit-S to ensure the accuracy and precision of the translation.

### 3.3. Data Collection Procedures

This study obtained approval from the Research Ethics Boards of the authors’ university. The participants provided informed consent before completing the study measures. A total of 306 participants completed an online questionnaire containing measures of the ILES and Grit-S. The time for answering the whole questionnaire lasted about 8 min.

### 3.4. Analysis Plan

We performed the analysis using JASP 0.17 (Love et al., 2019). Initially, we conducted preliminary analyses of the items and presented descriptive statistics. Subsequently, an exploratory factor analysis (EFA) was conducted to ascertain the factor loading and the underlying factor structure of the items. Although we have a well-established hypothetical model on engagement in language learning, its fitting with the interpreting learning needs further exploration, and the wordings in items were also changed. Therefore, we randomly separated the sample into two groups, with the first group being used for EFA. Following this, a confirmatory factor analysis was undertaken to reinforce the construct validity with the second group. Additionally, we utilized participants’ measurement of the personal trait of grit as a psychological factor to evaluate the external aspect of construct validity.

## 4. Results

### 4.1. Item-Level Analysis

The item-level analysis is presented in Table 1. The Skewness and Kurtosis values (all smaller than ±2) indicate that each item was nearly normally distributed (King & Caleon, 2021). The internal consistency was also confirmed, with all item–rest correlations larger than 0.5. For the Cronbach’s alpha, the overall engagement scale was 0.945. Each dimension also had adequate internal reliability on Cronbach’s alpha (BE: 0.904, EE: 0.891, CE: 0.887, and AGE: 0.875).

### 4.2. Exploratory Factor Analysis

To determine the appropriateness of conducting an EFA, a Kaiser–Meyer–Olkin (KMO) test was performed, and the results show that it was marvelous for the scale with a sampling adequacy value of 0.914 (>0.9). Bartlett’s test also shows that the data were appropriate for EFA (*χ*^2^ = 2224.504, *df* = 171.000, and *p* < 0.001).

**Figure 1:**
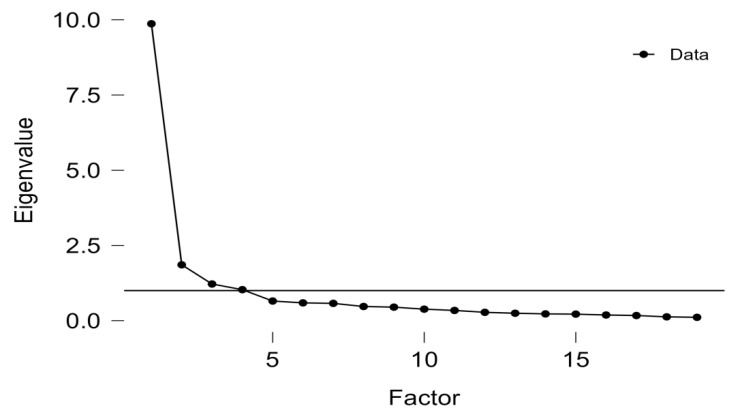
The scree plot.

From the scree plot (Figure 1), the optimum number of factors was four based on the criterion that the eigenvalues were above 1 and factoring the method of maximum likelihood. An orthogonal rotation (i.e., varimax) was performed to increase the solution. Both unrotated and rotated solution results show the cumulative of the four factors explained over 65% variance (see Table 2 for details). This means that the engagement scale effectively captures the four distinct aspects of interpreting learning engagement: behavioral, emotional, cognitive, and agentic. These factors together account for most of the variation in how students engage in interpreting learning. Table 3 shows the rotated factor pattern of the items. The overall pattern was in line with the hypothesized model, with four factors corresponding to the four dimensions of engagement. However, one item (EE1) had a factor loading of less than 0.5 and was a cross-loading item. Therefore, we decided to delete this item to increase precision and rerun the EFA. The whole result pattern was unchanged.

### 4.3. Confirmatory Factor Analysis

We performed a CFA model to testify that the four-factor model was derived from the EFA. The four-factor model showed satisfactory fit indices, with *χ*^2^ = 256.016, *df* = 129, *p* < 0.001, Comparative Fit Index (CFI): 0.938, Tucker–Lewis Index (TLI): 0.927, Bollen’s Incremental Fit Index (IFI): 0.939, Root Mean Square Error of Approximation (RMSEA): 0.080, and Standardized Root Mean Square Residual (SRMR): 0.059. This confirms that the four-factor model is a good representation of how students engage with interpreting learning tasks. The statistical fit suggests the scale works reliably across different samples of interpreting students. We linked residual covariances between CE2 and CE3 (z = 5.645, and *p* < 0.001) and CE4 and CE5 (z = 4.552, and *p* < 0.001). CE2 and CE3 were related because the two items measure the extent to which students pay attention to the teacher’s teaching (CE2) and other students responding (CE3), both associated with attention to others’ performance. CE4 and CE5 were correlated, possibly because both of them were about performance during the class. CE4 measures effort to perform well in the interpretation training class, and CE5 measures effort to engage fully in the class.

### 4.4. Correlation Between Interpreting Learning Engagement and Grit

We further explored the relationship between interpreting learning engagement with grit to establish the external validity. The correlation matrix is presented in Table 4. The results show that all four dimensions of interpreting learning engagement were positively related to grit and its two dimensions, namely, consistency of interest and perseverance of effort. This indicates that students who show more perseverance and passion for long-term goals (grit) are more likely to be actively and meaningfully engaged in interpreting learning.

## 5. Discussion

In this study, we developed and validated the Interpreting Learning Engagement Scale (ILES). Subsequently, we conducted an in-depth examination of the psychometric properties of this adapted scale. Our results indicate that the ILES exhibits adequate psychometric properties, and scores on the scale are significantly correlated with grit, a construct previously associated with language learning (Wang, 2021). Our findings fill an important gap in the literature by providing a validated tool tailored specifically for assessing student engagement in the interpreting learning context. We have included the full text of the ILES in both English and Chinese as Supplementary Materials.

Considering the interpreting learning environment, we modified the item wording to reflect the potential behavioral, emotional, cognitive, and agentic reactions of Chinese university students. The final ILES contained four factors corresponding to the four dimensions of engagement originally retrieved from the L2 Engagement Scale developed by Oga-Baldwin (2019). Factor 1, behavioral engagement, captured students’ expenditure of effort on learning tasks, the quality of their participation, and their degree of active involvement in the learning process (Sang & Hiver, 2021). Factor 2, cognitive engagement, captured students’ deliberate, selective, and sustained attention to achieve a given task or learning goal (Reeve, 2012; Svalberg, 2009). Factor 3, emotional engagement, captured students’ attitudes toward learning contexts, the members in that context, the learning tasks, and their own participation in learning (Reeve, 2012). Factor 4, agentic engagement, captured the extent to which students contribute agentically to the ongoing flow of the instruction they receive (Reeve & Tseng, 2011). These four factors provide a comprehensive framework for interpreting students’ engagement in learning. By focusing on these distinct dimensions, the ILES enables educators to pinpoint areas where students may require additional support. For instance, teachers can identify whether students struggle with maintaining cognitive focus, participating actively in class, or feeling emotionally connected to the learning context. Despite the change of item wording to fit the interpreting learning context, the ILES preserved core elements of engagement from the original L2 Engagement Scale and extended its applicability to the unique challenges and skills required in interpreting learning, such as real-time translation between languages, the need for memory retention, and the cognitive demands involved in interpreting tasks. These make interpreting learning distinct from other academic domains, and the ILES addresses these needs by focusing on the dimensions of engagement that are crucial for building interpreting competence.

The exploratory factor analysis (EFA) results reveal that the data from Chinese university students aligned well with the models proposed by the Interpreting Learning Engagement Scale (ILES). The ILES comprised eighteen items, with one item (EE1) removed due to cross-loading. Additionally, both the exploratory factor analysis (EFA) and confirmatory factor analysis (CFA) results consistently demonstrate the same factor structure across two distinct student samples. In essence, the CFA model corroborated the four-factor model derived from the EFA. In line with prior research exploring the relationship between domain-specific grit and engagement across various educational domains, such as L2 learning (Teimouri et al., 2022), academic productivity (Hodge et al., 2018), and nursing education (Sellers et al., 2019), our study also found significant correlations between the ILES scores and the two dimensions of grit. This validation underscores the external validity of the ILES.

Moreover, while previous studies have often highlighted correlations between grit and engagement in various learning contexts (Derakhshan & Fathi, 2024; O’Neal et al., 2019), they have not consistently distinguished between the components of grit in their association with learning engagement. However, our findings diverge from this trend. Contrary to the meta-analysis of Lam and Zhou (2022), which suggested a higher correlation between academic performance and perseverance of effort (PoE) compared to consistency of interest (CoI), our study found similar correlation coefficients between PoE, CoI, and interpreting learning engagement. This suggests that in the context of interpreting, a cognitively and affectively demanding skill, both PoE and CoI are crucial for the development of interpreting skills.

One implication of the present study is that we developed and validated the ILES to measure student engagement in the context of interpreting learning in China. The findings suggest that the 18-item measures are reliable and valid for Chinese university students learning interpreting. Student engagement drives learning (Gettinger & Ball, 2008). A better understanding of engagement can better equip educators to investigate how to engage learners (Philp & Duchesne, 2016). However, few valid instruments for interpreting learning engagement emerged compared with other domain-specific engagement scales. An accurate assessment of students’ interpreting learning engagement is crucial in helping students expend energy and effort, boost interest in participation, direct attentional resources in effective ways, and contribute to class interactions to support successful learning, and scaffolding should be provided to help students develop the capacity to act upon the demands associated with interpreting learning. Adapting and validating the existing L2 Engagement Scale with Chinese university students in the context of interpreting learning can provide information on the domain-specificity of engagement and shed light on potential ways to optimize students’ learning engagement for interpreting competence development.

Building on the ILES, educators can design targeted interventions to enhance student engagement, particularly when any of the four dimensions of engagement, i.e., cognitive, behavioral, emotional, or agentic, are measured to be lacking or declining at certain stages of interpreting learning. For instance, the introduction of problem-based learning (PBL) could prompt students to critically examine interpreting challenges and develop effective strategies for resolving them, thus fostering greater cognitive engagement. The implementation of metacognitive training could further encourage students to reflect on their learning processes, helping to improve cognitive involvement. To promote positive emotional engagement, cultivating a supportive learning environment where mistakes are viewed as a natural part of the learning process can help boost students’ confidence and motivation. In terms of behavioral engagement, incorporating gamified elements into interpreting exercises, such as setting challenges, creating leaderboards, or awarding points for task completion and accuracy, may enhance student participation and sustained effort during practice sessions. Finally, to empower agentic engagement, organizing regular individual feedback meetings where students can discuss their progress, challenges, and strategies for improvement will allow them to take an active role in adjusting their learning process and feel more in control. Future empirical studies will be required to evaluate the relative effectiveness of potential interventions and determine which strategies yield the most significant impact on students’ engagement in interpreting learning.

Another potential implication of this study lies in the potential applicability of the ILES beyond the Chinese context. While the scale was developed and validated with Chinese university students, its theoretical foundation, rooted in universal constructs of behavioral, cognitive, emotional, and agentic engagement, makes it adaptable to diverse cultural settings. Future studies could replicate this research to examine how cultural factors, shaped by societal norms and expectations, such as individualism versus collectivism or attitudes toward academic diligence and attainment, influence interpreting learning engagement in other countries compared to China. To facilitate cross-cultural application, researchers could translate and culturally adapt the ILES, ensuring that the language and context of the items are relevant to the target population and culture. Conducting cross-cultural validation studies would not only confirm the scale’s generalizability but also uncover potential cultural nuances in interpreting learning engagement.

There are several limitations to the study. First, we only recruited students learning interpreting with five language pairs from Mainland China. There is a need to replicate the research across diverse cultural and language contexts beyond China. Second, we validated the internal consistency of the ILES and its external consistency with another psychological construct, i.e., grit, but we did not probe into the potential correlation between interpreting learning engagement and academic performance. Specifically, further studies could explore how interpreting learning engagement links with other variables, e.g., students’ interpreting performance, learning motivation, and self-efficacy. Third, some theories of engagement also consider the social dimension (Heilporn et al., 2024), as reflected by a sense of belonging and connections with others during learning, although this dimension is not readily included in some previous attempts. Therefore, future studies could include this dimension accordingly. It is also worth noting that Heilporn et al. (2024) combined emotional and cognitive facets into the same scale. Thus, future studies could also compare and explore interpreting learning engagement in different theoretical frameworks. Finally, despite the advantages of self-report surveys and questionnaires highlighted in the literature and adopted in this study, there are still concerns about this approach to measuring engagement, such as potential participant bias leading to skewed results and the lack of real-time data. Future studies exploring interpreting learning engagement can incorporate external measures often used in language learning settings, such as observational instruments, to investigate the visible behaviors of individual students at multiple time points alongside survey instruments. Other external measures include eye-tracking to measure students’ gaze, attention, and time on task, biometric wearables measuring heart rate to give real-time measures of students’ reactions to classroom instruction and activities, and reaction times to triangulate retrospective and concurrent self-reports. These methods are likely to provide new measures of behavioral, emotional, cognitional, and agentic engagement and offer more accurate ways to model interpreting learning engagement constructs.

## 6. Conclusions

This study contributes to the literature by adapting and validating the L2 Engagement Scale to measure interpreting learning engagement in Chinese settings. The findings suggest that the ILES measures are reliable and valid for university students learning interpreting.

## Figures and Tables

**Table 1:** Descriptive statistics for the Interpreting Learning Engagement Scale.

	Mean	SD	Skewness	Kurtosis	Item–Rest Correlation
BE1	4.141	0.694	−0.551	0.410	0.728
BE2	4.160	0.690	−0.583	0.508	0.709
BE3	3.892	0.792	−0.642	0.704	0.684
BE4	4.000	0.793	−0.634	0.413	0.693
BE5	3.951	0.798	−0.730	0.846	0.695
EE1	3.614	0.986	−0.570	0.054	0.683
EE2	3.961	0.867	−0.561	−0.016	0.769
EE3	3.987	0.802	−0.553	0.173	0.705
EE4	4.131	0.819	−0.786	0.571	0.729
CE1	3.990	0.800	−0.602	0.499	0.734
CE2	4.026	0.785	−0.743	0.945	0.656
CE3	4.013	0.772	−0.839	1.330	0.640
CE4	4.023	0.795	−0.829	1.085	0.689
CE5	4.023	0.753	−0.780	1.111	0.661
AGE1	3.676	0.911	−0.416	−0.348	0.589
AGE2	3.278	1.042	−0.034	−0.694	0.603
AGE3	3.641	0.996	−0.373	−0.575	0.638
AGE4	3.356	1.059	−0.135	−0.658	0.626
AGE5	3.474	1.009	−0.285	−0.569	0.643

Note: BE: behavioral engagement, EE: emotional engagement, CE: cognitive engagement, AGE: agentic engagement.

**Table 2:** The proportion of variance explained by the factors.

	Unrotated Solution		Rotated Solution	
	Proportion of Variance	Cumulative	Proportion of Variance	Cumulative
Factor 1	0.502	0.502	0.190	0.190
Factor 2	0.080	0.582	0.187	0.377
Factor 3	0.049	0.631	0.149	0.526
Factor 4	0.039	0.670	0.142	0.668

**Table 3:** Rotated factor loadings.

	Factor 1	Factor 2	Factor 3	Factor 4
BE1	0.821			
BE2	0.807			
BE3	0.668			
BE4	0.608			
BE5	0.603			
EE1	0.411	0.499		
EE2				0.682
EE3				0.810
EE4				0.751
CE1			0.610	
CE2			0.638	
CE3			0.720	
CE4			0.571	
CE5			0.560	
AGE1		0.635		
AGE2		0.629		
AGE3		0.660		
AGE4		0.820		
AGE5		0.798		

Note: BE: behavioral engagement, EE: emotional engagement, CE: cognitive engagement, AGE: agentic engagement.

**Table 4:** Correlation matrix of interpretation learning engagement and grit.

	1	2	3	4	5	6	7
CoI	-----						
PoE	0.436 ***	-----					
Grit	0.840 ***	0.855 ***	-----				
BE	0.267 ***	0.260 ***	0.311 ***	-----			
EE	0.205 ***	0.224 ***	0.253 ***	0.690 ***	-----		
CE	0.224 ***	0.225 ***	0.265 ***	0.708 ***	0.678 ***	-----	
AGE	0.226 ***	0.229 ***	0.269 ***	0.548 ***	0.543 ***	0.566 ***	-----

Note: *** *p* < 0.001, CoI: consistency of interest, PoE: perseverance of effort, BE: behavioral engagement, EE: emotional engagement, CE: cognitive engagement, AGE: agentic engagement.

## Data Availability

The data are available from the corresponding author upon reasonable request.

## Supplementary Materials

The following supporting information can be downloaded at: https://www.mdpi.com/article/10.3390/bs15010016/s1, Table S1: ILES (English); Table S2: ILES (Chinese).
