# Supplementary material for: Development and Validation of the Interpreting Learning Engagement Scale (ILES)
Source: Behav Sci (Basel). 2024 Dec 28;15(1):16. doi: 10.3390/bs15010016 (PMC11759842; doi:10.3390/bs15010016)
Supplement: Supplementary file 1 [file behavsci-15-00016-s001.zip › behavsci-3303198-supplementary.pdf]

Supplementary Materials

Supplementary Table S1: ILES (English)

No.	Sub- construct	ILES Items
1	BE1	I participated in the activities of this semester's interpreting classes.
2	BE2	I paid attention to my interpreting teacher this semester.
3	BE3	I listened very carefully in the interpreting classes this semester.
4	BE4	I tried to do well in this semester's interpreting classes.
5	BE5	I worked as hard as I could in this semester's interpreting classes.
6	EE2	I enjoyed this semester's interpreting classroom activities.
7	EE3	I was interested in the materials of this semester's interpreting classes.
8	EE4	This semester's interpreting classes were fun.
9	CE1	I applied the interpreting skills and techniques that I learned in class to practising interpreting this semester.
10	CE2	I tried to fully comprehend the exact messages in the source speeches in this semester's interpreting classes.
11	CE3	I tried to fully deliver the exact messages in the target Language in this semester's interpreting classes.
12	CE4	I thought about the teacher's and peer students' feedback on my interpreting performance in this semester's interpreting classes.
13	CE5	I thought about the strengths and weaknesses of my interpreting performance in this semester's interpreting classes.
14	AGE1	I let my teacher know what I considered difficult in practising interpreting in this semester's interpreting classes.
15	AGE2	I let my teacher know what I was satisfied with the most about my interpreting practice in this semester's interpreting classes.
16	AGE3	I expressed my opinions in the interpreting classes this semester.
17	AGE4	I asked questions to help myself learn more knowledge and skills of interpreting in this semester's interpreting classes.
18	AGE5	When I ran into difficulties in practising interpreting in this semester's interpreting classes, I asked my teacher for advice.

Supplementary Table S2: ILES (Chinese)

No.	Sub- construct	ILES Items
1	BE1	我认真参与了这学期口译课的课堂口译学习。
2	BE2	我在这学期口译课上专心地听老师讲课。
3	BE3	我在这学期口译课上仔细听其他同学发言。
4	BE4	我在这学期口译课上努力表现好。
5	BE5	我在这学期口译课上尽我所能投入学习。
6	EE2	我很喜欢参与这学期口译课的课堂活动。
7	EE3	我对这学期口译课的教学材料感兴趣。
8	EE4	这学期的口译课很有趣。
9	CE1	这学期口译课上的练习我运用了所教授的口译技巧和策略。
10	CE2	这学期口译课上我尝试充分理解源语中的确切信息。
11	CE3	这学期口译课上我尝试用目标语充分译出源语的确切信息。
12	CE4	这学期口译课上我就老师和同学对我课上的口译表现的反馈进行了思考。

13	CE5	这学期口译课上我就自己在课上的口译表现的优缺点进行了思考。
14	AGE1	这学期我让老师知道我在课上练习口译时遇到哪些困难。
15	AGE2	这学期我让老师知道我在课上练习口译时自己感到最满意的部分。
16	AGE3	这学期口译课上我表达过自己的想法。
17	AGE4	这学期我通过提问来帮助自己学得更多口译相关的知识和技能。
18	AGE5	这学期当我在口译练习中遇到困难时，我会主动求助于老师。
